# Effects of Aging on Collagen in the Skeletal Muscle of Mice

**DOI:** 10.3390/ijms241713121

**Published:** 2023-08-23

**Authors:** Yuji Kanazawa, Ryo Miyachi, Takashi Higuchi, Hiaki Sato

**Affiliations:** 1 Department of Physical Therapy, Hokuriku University, Kanazawa 920-1180, Ishikawa, Japan; ry_miyachi@hokuriku-u.ac.jp; 2 Department of Physical Therapy, Osaka University of Human Sciences, Settsu 566-8501, Osaka, Japan; t.higuchi1124@gmail.com; 3 Department of Medical Technology and Clinical Engineering, Hokuriku University, Kanazawa 920-1180, Ishikawa, Japan; h-sato@hokuriku-u.ac.jp

**Keywords:** aging, skeletal muscle, collagen, slow-twitch muscle, fast-twitch muscle

## Abstract

Aging affects several tissues in the body, including skeletal muscle. Multiple types of collagens are localized in the skeletal muscle and contribute to the maintenance of normal muscle structure and function. Since the effects of aging on muscle fibers vary by muscle fiber type, it is expected that the effects of aging on intramuscular collagen might be influenced by muscle fiber type. In this study, we examined the effect of aging on collagen levels in the soleus (slow-twitch muscle) and gastrocnemius (fast-twitch muscle) muscles of 3-, 10-, 24-, and 28-month-old male C57BL/6J mice using molecular and morphological analysis. It was found that aging increased collagen I, III, and VI gene expression and immunoreactivity in both slow- and fast-twitch muscles and collagen IV expression in slow-twitch muscles. However, collagen IV gene expression and immunoreactivity in fast-twitch muscle were unaffected by aging. In contrast, the expression of the collagen synthesis marker heat shock protein 47 in both slow- and fast-twitch muscles decreased with aging, while the expression of collagen degradation markers increased with aging. Overall, these results suggest that collagen gene expression and immunoreactivity are influenced by muscle fiber type and collagen type and that the balance between collagen synthesis and degradation tends to tilt toward degradation with aging.

## 1. Introduction

Skeletal muscle tissue is composed of muscle fibers, which are multinucleated cells and can be classified into the slow- and fast-twitch types [1]. Skeletal muscle is responsible for physiological processes, including postural maintenance, joint movement, heat production, metabolism, and secretion of numerous peptides for communication with other tissues [1,2,3,4]. Therefore, it is critical to promote and maintain normal skeletal muscle structure and function for health. Aging adversely affects skeletal muscles. For example, sarcopenia is an aging-related condition characterized by progressive and generalized loss of skeletal muscle mass and strength and correlates with physical disability and decreased quality of life [5,6]. Pathological changes in sarcopenia include decreased muscle protein synthesis and muscle atrophy [5,7,8,9,10]. Furthermore, aging-related muscle atrophy occurs primarily in fast-twitch muscles [7,8,9,10]. Therefore, differences in muscle fiber type must be considered to understand the effects of aging on skeletal muscle.

Muscle fibers are surrounded by an extracellular matrix (ECM) consisting of a mesh of collagen components and a mixture of macromolecules, such as various glycoproteins and proteoglycans [11]. The ECM plays an important role in muscle development, growth, and regeneration, as well as in the transmission of contractile force [12,13,14,15,16,17]. Fibroblasts are representative cells that produce ECM proteins, including collagen, in skeletal muscle and contribute to ECM construction [18]. Fibroblasts produce various types of collagens, including collagen I and III, the major collagens in skeletal muscle, and collagen IV and VI, which localize to the basement membrane (BM) of the muscle tissue [18]. The collagen-producing capacity of fibroblasts is also influenced by aging [19]. Additionally, the aging-related decrease in collagen production delays recovery from muscle atrophy or injury [20,21]. Therefore, suppressing the effects of aging on collagen production in skeletal muscle can contribute to the maintenance of normal skeletal muscle structure and function.

Individual skeletal muscles are surrounded by multiple layers of ECMs, named epimysium, perimysium, endomysium, and BM [11]. The BM and endomysium are the innermost layers that surround the individual muscle fibers, the perimysium binds the group of muscle fibers, and the epimysium surrounds the entire muscle. The most abundant collagens in skeletal muscle are collagens I and III, which are fibrous collagens localized in the epimysium, perimysium, and endomysium [17,22,23,24]. Non-fibrous collagens, including collagen IV and VI, localize to the BM [15,25]. Collagen IV is more concentrated in slow-twitch muscles than fast-twitch muscles [26]. Therefore, the effect of aging on collagen in skeletal muscle is expected to vary by muscle fiber type. However, several unresolved issues exist regarding the differences in aging-induced changes in collagen expression with respect to skeletal muscle fiber and collagen types. In this study, we compared the effects of aging on multiple collagen types in slow- and fast-twitch muscles of mice using morphological and molecular analyses. In addition, the aging process was examined in mature adult (3 months old, 3 M), middle-aged (10 months old, 10 M), and old (24 [24 M] and 28 months [28 M]) mice in a time course study. The time points selected corresponded to the 20–30, 38–47, and 56–69 years of human life [27]. Therefore, the findings of this study are expected to provide basic data that could be considered for future application to humans.

## 2. Results

### 2.1. Body Weight, Muscle Weight, Relative Muscle Weight, and Fiber Cross-Sectional Area

**Figure 1:**
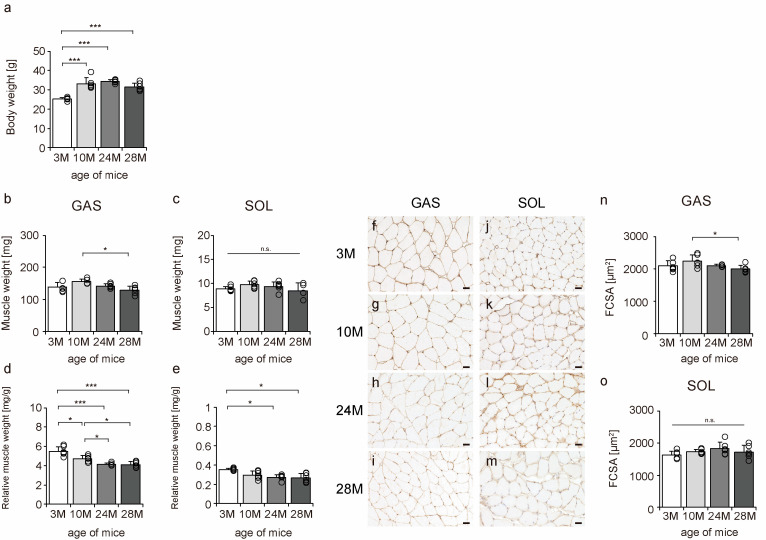
Changes in body weight, muscle weight, relative muscle weight, and fiber cross-sectional area. Body weight (**a**), muscle weight (**b**,**c**), relative muscle weight (**d**,**e**), cross-sections stained with an anti-collagen IV antibody (**f**–**m**), and fiber cross-sectional area (FCSA) (**n**,**o**). Staining of the gastrocnemius (**f**–**i**) and soleus (**j**–**m**) muscles observed in 3 M (**f**,**j**), 10 M (**g**,**k**), 24 M (**h**,**l**), and 28 M (**i**,**m**) for FCSA measurements (scale bar = 25 µm). Data are presented as the mean ± standard deviation (*n* = 6 per group). GAS, gastrocnemius muscle; SOL, soleus muscle; 3 M, 3-month-old; 10 M, 10-month-old; 24 M, 24-month-old; 28 M, 28-month-old. *** *p <* 0.0001, * *p <* 0.05. n.s.: not significant. All data are represented as dots in the graph.

To confirm the effects of aging on mouse skeletal muscle, body weight, muscle weight, relative muscle weight, and fiber cross-sectional area (FCSA) were measured. Body weight significantly increased from 3 M to 10 M. The body weight was approximately the same in 10 M and 24 M, but there was a slight downward trend in 28 M, although the differences were not statistically significant (Figure 1a). The gastrocnemius, a fast-twitch muscle, tended to atrophy more than the soleus muscle, a slow-twitch muscle (Figure 1b,c,n,o), a typical phenomenon of skeletal muscle aging [7,8,9,10]. Additionally, there was a decrease in the relative muscle weights of both slow- and fast-twitch muscles, confirming the aging phenomenon in both muscles (Figure 1d,e). These data confirmed aging-induced changes in the skeletal muscles of mice.

### 2.2. Expression of Collagen Genes

**Figure 2:**
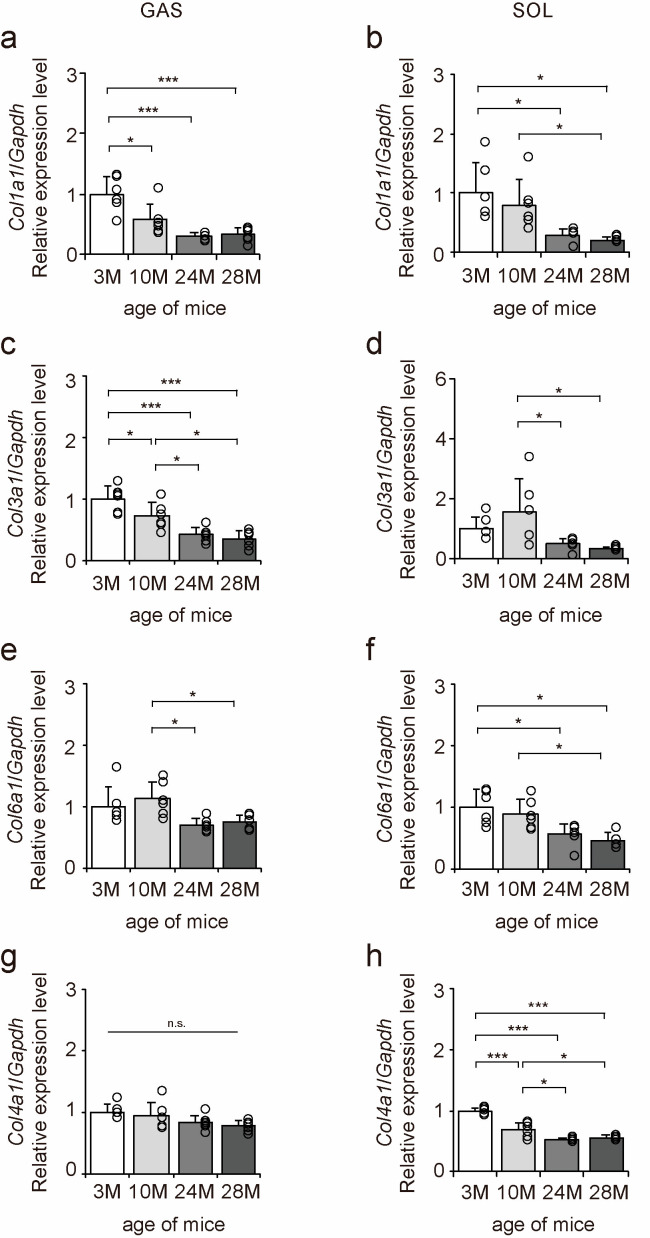
Changes in the expression of collagen genes in skeletal muscle. Changes in relative mRNA expression levels of *Col1a1* (**a**,**b**), *Col3a1* (**c**,**d**), *Col6a1* (**e**,**f**), and *Col4a1* (**g**,**h**) in the gastrocnemius (GAS) and soleus (SOL) muscles. Data are presented as the mean ± standard deviation, *n* = 6 per group. 3 M, 3-month-old; 10 M, 10-month-old; 24 M, 24-month-old; 28 M, 28-month-old. *** *p <* 0.0001, * *p <* 0.05. n.s.: not significant. All data are represented as dots in the graph.

The mRNA expression levels of collagen genes in the gastrocnemius and soleus muscles were examined to elucidate the effects of aging on collagen in skeletal muscle using quantitative PCR (qPCR) (Figure 2a–h). *Col1a1*, *Col3a1*, and *Col6a1* expression levels decreased with aging in both gastrocnemius and soleus muscles (Figure 2a–f). *Col4a1* expression level decreased with aging in the soleus muscle but was unaffected by aging in the gastrocnemius muscle (Figure 2g,h). Overall, these results suggest that aging caused a decrease in *Col1a1*, *Col3a1*, and *Col6a1* expression in both fast- and slow-twitch muscles and a decrease in *Col4a1* expression in only slow-twitch muscles.

### 2.3. Collagen I Localization

**Figure 3:**
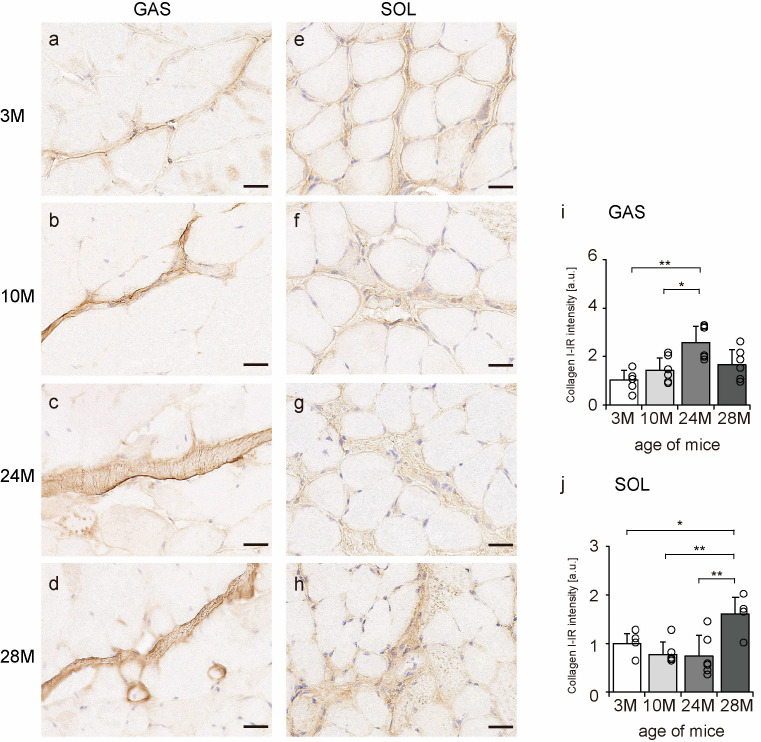
Collagen I localization. Cross-sections of the gastrocnemius (**a**–**d**) and soleus (**e**–**h**) muscles were stained with an anti-collagen I antibody. Staining images of the 3 M (**a**,**e**), 10 M (**b**,**f**), 24 M (**c**,**g**), and 28 M (**d**,**h**) groups are shown (scale bar = 25 µm). Collagen I-immunoreactivity (IR) intensity in gastrocnemius (**i**) and soleus muscles (**j**) was measured. Data are presented as the mean ± standard deviation, *n* = 6 per group. 3 M, 3-month-old; 10 M, 10-month-old; 24 M, 24-month-old; 28 M, 28-month-old; GAS, gastrocnemius muscle; SOL, soleus muscle. ** *p <* 0.001, * *p <* 0.05. All data are represented as dots in the graph.

Collagen I is a significant component of the endomysium, perimysium, and epimysium layers [11]. Transverse sections of skeletal muscle were observed to confirm the localization of collagen I using an immunohistochemical assay. In all the groups, collagen I immunoreactivity (IR) was confirmed in the endomysium and perimysium of the gastrocnemius and soleus muscles (Figure 3a–h). Additionally, collagen I-IR intensity increased with aging in the gastrocnemius muscle but showed a quadratic pattern in soleus muscles, decreasing in the 10 M and 24 M groups and increasing significantly in the 28 M group compared with that in the 3 M group (Figure 3i,j). These results suggest that aging increases collagen I concentration in both slow- and fast-twitch muscles.

### 2.4. Collagen III Localization

**Figure 4:**
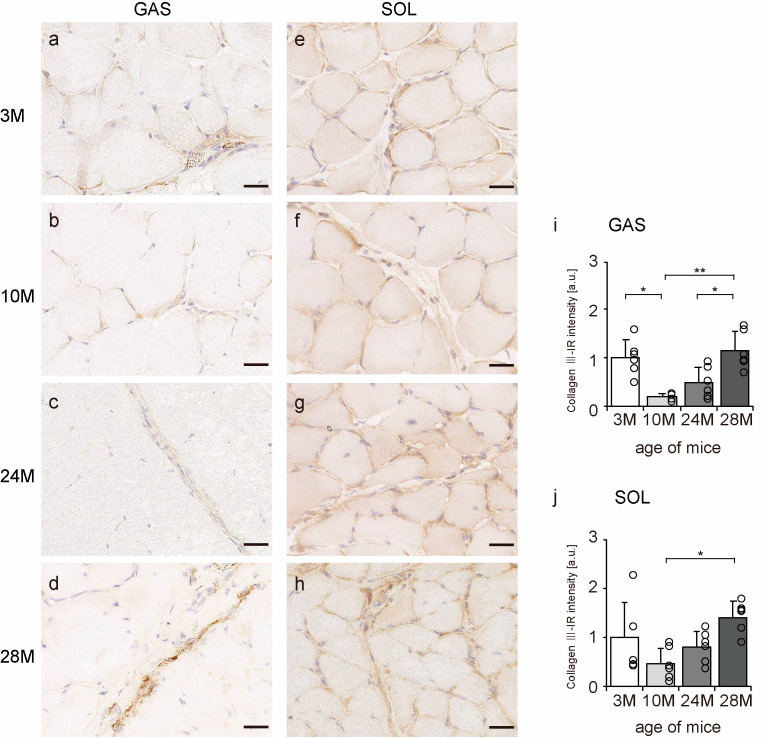
Collagen III localization. Cross-sections of the gastrocnemius (**a**–**d**) and soleus (**e**–**h**) muscles were stained with anti-collagen III antibody. Staining images of the 3 M (**a**,**e**), 10 M (**b**,**f**), 24 M (**c**,**g**), and 28 M (**d**,**h**) groups are shown (scale bar = 25 µm). Collagen III-immunoreactivity (IR) intensity in gastrocnemius (**i**) and soleus muscles (**j**) was measured. Data are presented as the mean ± standard deviation, *n* = 6 per group. 3 M, 3-month-old; 10 M, 10-month-old; 24 M, 24-month-old; 28 M, 28-month-old; GAS, gastrocnemius muscle; SOL, soleus muscle. ** *p <* 0.001, * *p <* 0.05. All data are represented as dots in the graph.

Collagen III is mainly localized in the endomysium and perimysium [11]. Transverse sections of skeletal muscle were observed to confirm the localization of collagen III using an immunohistochemical assay. In all the groups, collagen III-IR presence was confirmed in the endomysium and perimysium of the gastrocnemius and soleus muscles (Figure 4a–h). Additionally, the intensity of collagen III staining showed a quadratic pattern, decreasing in the 10 M and 24 M groups and increasing significantly in the 28 M group in both gastrocnemius and soleus muscles compared with the intensity in the 3 M group (Figure 4i,j). These results indicate that aging increases collagen III expression in both slow- and fast-twitch muscles.

### 2.5. Collagen VI Localization

**Figure 5:**
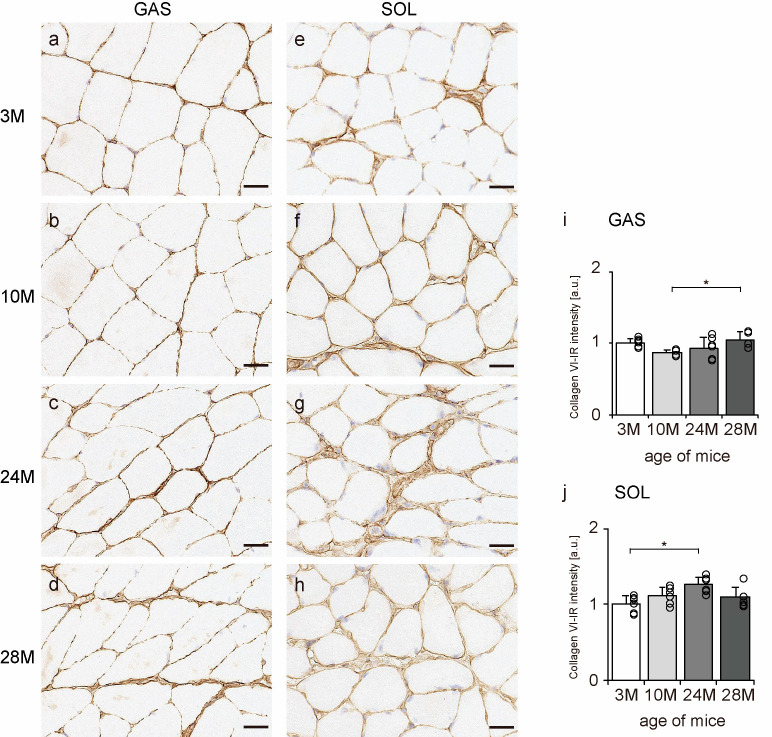
Collagen VI localization. Cross-sections of the gastrocnemius (**a**–**d**) and soleus (**e**–**h**) muscles were stained with anti-collagen VI antibody. Staining images of the 3 M (**a**,**e**), 10 M (**b**,**f**), 24 M (**c**,**g**), and 28 M (**d**,**h**) groups are shown (scale bar = 25 µm). Collagen VI-immunoreactivity (IR) intensity was measured in gastrocnemius (**i**) and soleus muscles (**j**). Data are presented as the mean ± standard deviation, *n* = 6 per group. 3 M, 3-month-old; 10 M, 10-month-old; 24 M, 24-month-old; 28 M, 28-month-old; GAS, gastrocnemius muscle; SOL, soleus muscle. * *p <* 0.05. All data are represented as dots in the graph.

Collagen VI is widely localized in endomysium, perimysium, and epimysium, but especially near the BM [11]. Therefore, transverse sections of skeletal muscle were observed to confirm the localization of collagen VI using an immunohistochemical assay. In all the groups, collagen VI-IR confirmed its expression in the BM, endomysium, and perimysium of the gastrocnemius and soleus muscles (Figure 5a–h). Additionally, the intensity of collagen VI staining increased with aging in both gastrocnemius and soleus muscles (Figure 5i,j), indicating that aging increased collagen VI expression in both slow- and fast-twitch muscles.

### 2.6. Collagen IV Localization 

**Figure 6:**
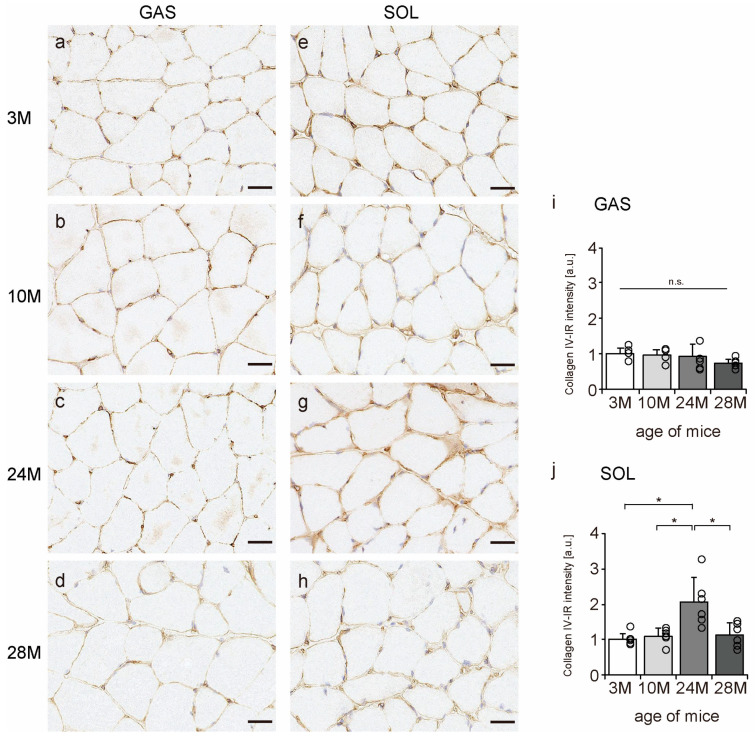
Collagen IV localization. Cross-sections of the gastrocnemius (**a**–**d**) and soleus (**e**–**h**) muscles were stained with anti-collagen IV antibody. Staining images of the 3 M (**a**,**e**), 10 M (**b**,**f**), 24 M (**c**,**g**), and 28 M (**d**,**h**) groups are shown (scale bar = 25 µm). Collagen IV-immunoreactivity (IR) intensity in gastrocnemius (**i**) and soleus muscles (**j**) was measured. Data are presented as the mean ± standard deviation, *n* = 6 per group. 3 M, 3-month-old; 10 M, 10-month-old; 24 M, 24-month-old; 28 M, 28-month-old; GAS, gastrocnemius muscle; SOL, soleus muscle. * *p <* 0.05. n.s.: not significant. All data are represented as dots in the graph.

Collagen IV is a significant component of the BM [11]; therefore, we confirmed whether collagen IV is localized in the BM using an immunohistochemical assay. In all the groups, collagen IV was expressed in the BM of the muscle fibers (Figure 6a–h). Additionally, collagen IV-IR intensity increased with aging in the soleus muscle but not in gastrocnemius (Figure 6i,j), suggesting that aging increased collagen IV expression in slow-twitch muscles but not in fast-twitch muscles.

### 2.7. Collagen Synthesis Marker

**Figure 7:**
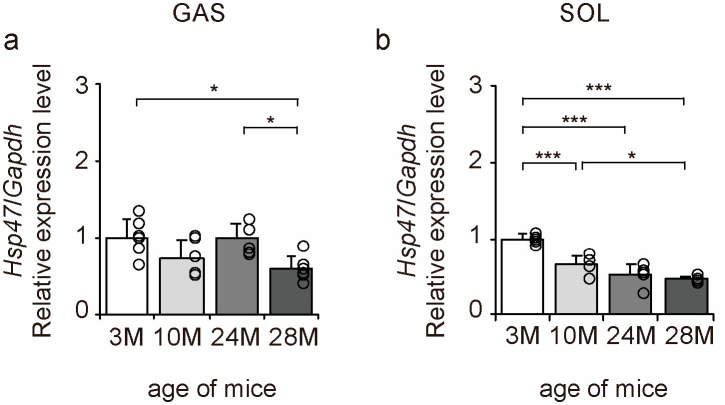
Changes in collagen synthesis marker expression in skeletal muscle. Changes in relative mRNA expression level of heat shock protein 47 (*Hsp47)* (**a**,**b**) in the gastrocnemius (GAS) and soleus (SOL) muscles. Data are presented as mean ± standard deviation, *n* = 6 per group. 3 M, 3-month-old; 10 M, 10-month-old; 24 M, 24-month-old; 28 M, 28-month-old. *** *p <* 0.0001, * *p <* 0.05. All data are represented as dots in the graph.

Heat shock protein 47 (HSP47) is a factor responsible for collagen folding in cells [28]. Therefore, we examined the expression of *Hsp47* to elucidate the molecular mechanisms underlying the effects of aging on collagen synthesis using qPCR (Figure 7a,b). Aging significantly decreased the expression of the collagen synthesis marker *Hsp47* in gastrocnemius and soleus muscles (Figure 7a,b). These results suggest that collagen synthesis declines with age in both slow- and fast-twitch muscles in a steady state.

### 2.8. Collagen Degradation Markers

**Figure 8:**
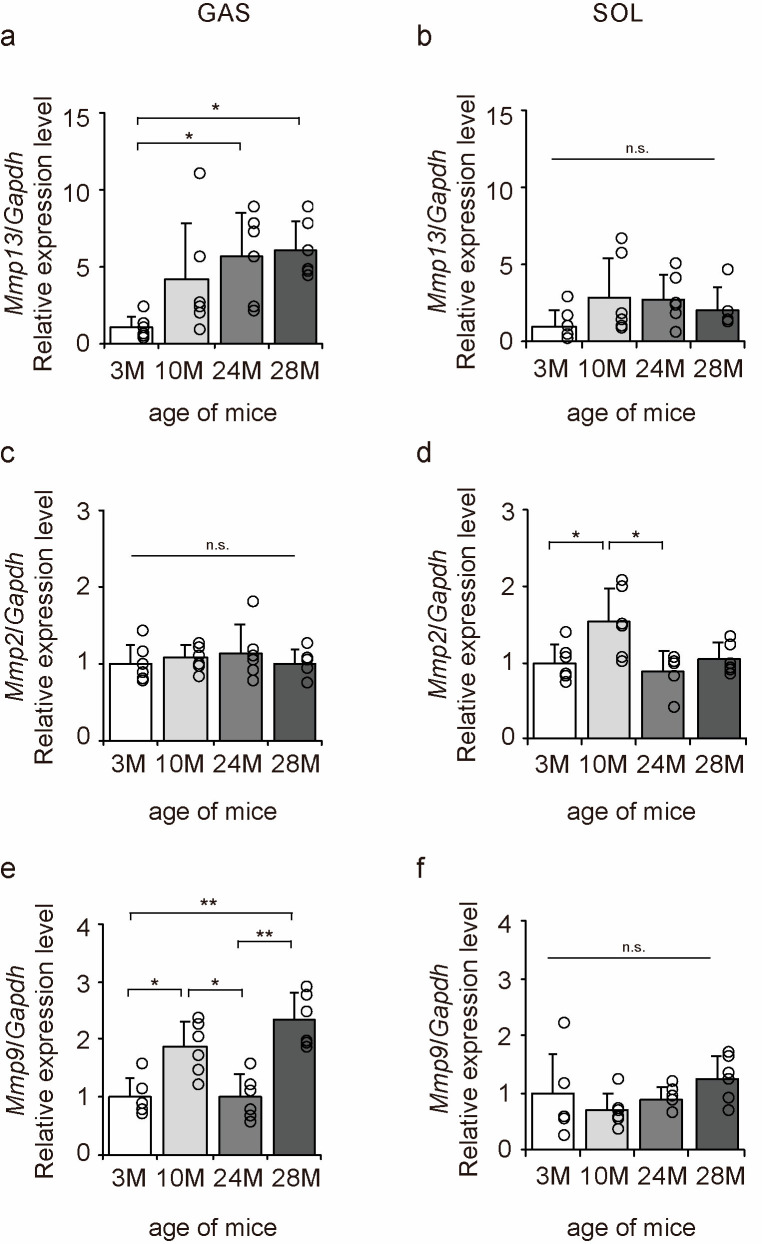
Changes in the expression of collagen degradation factors in the skeletal muscle. Changes in relative mRNA expression levels of matrix metalloproteinase 13 (*Mmp13*) (**a**,**b**), *Mmp2* (**c**,**d**), and *Mmp9* (**e**,**f**) in the gastrocnemius (GAS) and soleus (SOL) muscles. Data are presented as the mean ± standard deviation (*n* = 6 per group). 3 M, 3-month-old; 10 M, 10-month-old; 24 M, 24-month-old; 28 M, 28-month-old. ** *p <* 0.001, * *p <* 0.05. n.s.: not significant. All data are represented as dots in the graph.

Matrix metalloproteinases (MMPs) are enzymes responsible for the degradation of collagen [29]. MMP13 is a degradation marker for fibrotic collagen, while MMP2 and MMP9 are degradation markers for non-fibrotic collagen [29]. Therefore, we examined the expression of *Mmp13*, *Mmp2*, and *Mmp9* to elucidate the molecular mechanisms underlying the effects of aging on collagen degradation using qPCR (Figure 8a–f). Aging significantly increased *Mmp13* and *Mmp9* expression in the gastrocnemius muscle (Figure 8a,e) and *Mmp2* expression in the soleus muscle (Figure 8d). These results suggest that the collagen-degradative response increases with aging in both slow- and fast-twitch muscles.

## 3. Discussion

In the present study, we examined the effects of aging on collagen in the skeletal muscles of mice. qPCR showed that aging decreased the expression of *Col1a1*, *Col3a1*, and *Col6a1* in both slow- and fast-twitch muscles and *Col4a1* expression in only slow-twitch muscle. Additionally, the immunohistochemical assay showed that collagen I, III, and VI-IR increased in both slow- and fast-twitch muscles and collagen IV-IR increased only in slow-twitch muscle with aging. Moreover, aging decreased the expression of the collagen synthesis marker *Hsp47* but increased the expression of *Mmps*, which are collagen degradation markers, in skeletal muscle. The results also suggested that collagen IV gene expression in fast-twitch muscle may be less affected by aging.

Aging-induced decrease in collagen gene expression may vary with collagen type and muscle fiber type. A previous in vitro study showed a decrease in collagen I gene expression in aging fibroblasts [19]. Additionally, another study reported a decrease in *Col1a1* and *Col3a1* expression in skeletal muscle in vivo at 20–24 months of age compared with the expression level at 3–5 months in mice [30]. Thus, the observed decrease in *Col1a1*, *Col3a1*, and *Col6a1* expression in both slow- and fast-twitch muscles in the present study was consistent with previous findings. However, the effect of aging on *Col4a1* expression differed by muscle fiber type. A previous study reported lower *Col4a1* expression in rat plantaris muscle at 20 months of age than at 3 months [31]. Consistent with the present findings, *Col4a1* expression was unaffected by aging in the gastrocnemius muscle of mice fed a normal diet [32]. The soleus and plantaris muscles have stronger slow-twitch muscle characteristics than the gastrocnemius muscle [33,34]. Moreover, collagen IV content is reported to be higher in slow-twitch muscles than in fast-twitch muscles, and aging and training further affect the composition of the BM, including collagen IV expression, in slow-and fast-twitch muscles [26]. Therefore, collagen IV gene induction may be more influenced by muscle fiber type than other collagen genes.

In the present study, collagen I, III, and VI-IR intensity in slow- and fast-twitch muscles and collagen IV-IR intensity in slow-twitch muscle increased with aging. However, collagen IV-IR in fast-twitch muscle was not significantly affected by aging, indicating that the accumulation of collagen in skeletal muscle with aging can vary with collagen type and muscle fiber type. Previous studies have reported an increase in collagen I expression with aging in the skeletal muscles of mice and humans [35] and an increase in collagen I and III expression with aging in the gastrocnemius muscle of mice [36]. Additionally, collagen VI expression increased with aging in the myocardium of mice [37]. These findings suggest that fibrous and beaded collagen may accumulate with aging, regardless of muscle fiber type. In contrast, the effect of aging on collagen IV-IR varied by muscle fiber type in the present study. Previous studies have shown that aging increased collagen IV content in the skeletal muscle of mice [38,39] and increased the accumulation of collagen IV in human cerebral micro-vessels [40]. Since collagen IV is also produced by pericytes in micro-vessels [41], the accumulation of collagen IV with aging may be more pronounced in the soleus muscle, which is rich in micro-vessels, than in the gastrocnemius muscle. Additionally, it has been reported that collagen accumulation with aging is related to changes in the environment surrounding fibroblasts [42]. Mechanical stress stimulates ECM remodeling by fibroblasts, but mechanical stress is not transmitted to fibroblasts in aging tissues, resulting in collagen accumulation [42]. Although the reason for the lack of age-related collagen IV accumulation in the gastrocnemius muscle in this study is unclear, it is possible that since collagen IV is primarily localized in the BM [25], ECM remodeling was limited to the BM of the gastrocnemius muscle, which is less susceptible to age-related collagen accumulation than the soleus muscle.

Furthermore, *Hsp47* expression decreased with aging in both slow- and fast-twitch muscles in the present study, suggesting a decrease in collagen synthesis with aging in these muscles. Similarly, an age-related decrease in *Hsp47* expression has been observed in the plantaris muscle, which has both slow- and fast-twitch muscle characteristics [31]. Moreover, an age-related decrease in *Hsp47* expression was also observed in fibroblasts in an in vitro study [43]. These findings suggest that the age-related decline in *Hsp47* induction occurs in both slow and fast-twitch muscles. HSP47 is localized within the rough endoplasmic reticulum, where it produces collagen using procollagen [28]. Moreover, *Hsp47* expression correlates with various types of collagens [28]. Thus, the aging-induced decrease in *Hsp47* expression in this study is expected, as the gene expression of multiple collagens decreased in both slow- and fast-twitch muscles.

In the present study, aging generally increased the expression of MMP genes in skeletal muscle. MMP13 is an enzyme that degrades fibrous collagen, mainly collagen I and III [44], and has also been reported to contribute to growth and regeneration in skeletal muscle [45]. A previous in vitro study using chondrocytes reported increased expression of MMP13 with aging [46]. Additionally, MMP2 and MMP9 are gelatinases that use collagen IV as a substrate [47], and their expression has been reported to increase with aging in mouse gastrocnemius, soleus, and cardiac muscles [48,49,50]. In the present study, there was a general trend toward accelerated degradation of collagen I, III, VI, and VI in skeletal muscle, which may have been influenced by the fact that the mechanical stimulation of fibroblasts decreases with aging. In the aging human skin dermis, the binding sites of collagen fibers are lost, and mechanical resistance to traction forces is reduced [51,52]. Decreased mechanical stress has also been reported to promote the induction of MMPs in fibroblasts [51,52]. Furthermore, it has been reported that the expression of tissue inhibitors of MMPs (TIMPs), physiological inhibitors of MMPs, is decreased in the Achilles tendons of older rats, while MMP expression and activity are excessive [53]. Decreased TIMP expression might be responsible for the increase in MMPs in skeletal muscle with aging. Despite the enhanced induction of *Mmps*, collagen degraders, and multiple types of collagens accumulated in skeletal muscle in the present study, which may be attributed to age-related collagen modification. Previous studies have reported that aging induces collagen modification via mineralization, accumulation of advanced glycation end products, and depletion of glycosaminoglycans, which affects fiber stability and susceptibility to MMP-mediated degradation [54]. Moreover, collagen modification is known to cause a decrease in the turnover of collagen [55]. Thus, the simultaneous increase in MMP expression and collagen accumulation with aging may be part of the aging process in skeletal muscle.

Although interesting findings were discovered in this study, there are some limitations. First, this study examined the effects of aging on intramuscular collagen in skeletal muscle only at a steady state. Previous studies have reported that gene induction and IR of intramuscular collagen fluctuate with recovery after muscle atrophy or injury, high-fat diet intake, and stretching [20,21,32,56]. Therefore, further analysis of the effects of aging on the reactivity of intramuscular collagen production and degradation at different physiological states is required. Second, this study only confirmed the effect of aging on MMPs at the transcriptional level; MMPs degrade collagen as a substrate when activated from pro-MMPs to activated MMPs [57]. Therefore, it is thought that the effect of aging on skeletal muscle collagen can be better understood by examining the post-transcriptional activation of MMPs. Third, the data obtained in this study are limited to qPCR and immunohistochemical imaging, and the detailed mechanisms of the effects of aging on collagen remain unresolved. In particular, collagen-IR intensity increased with aging. However, transcripts and the collagen synthesis marker decreased, while degradation markers increased. This leaves us with an unclear understanding of the underlying mechanism behind the increase in collagen-IR intensity. Furthermore, immunohistochemical analysis confirmed the intramuscular localization of collagen and the IR intensity in the entire muscle cross section. Yet, no analysis has been conducted to determine whether the increased collagen levels are primarily present in the perimysium or endomysium. Additionally, it remains unknown whether this collagen increase is centered around fast-twitch or slow-twitch muscle fibers. Further in-depth analysis of collagen localization within skeletal muscle, as well as quantitative assessments using hydroxyproline assays and protein expression measurements, are required to elucidate the exact mechanism. Fourth, in this study, we showed that collagen in skeletal muscle changes with aging according to muscle fiber type, but the reasons and detailed mechanisms through which aging affects collagen production remain unresolved. In particular, it is unclear whether the decline in fibroblast function is directly responsible for skeletal muscle aging. Interestingly, it has been reported that knockout of transcription factor 4 (TCF4), which is highly expressed in the fibroblasts of intramuscular connective tissue, results in insufficient recovery of regenerating muscles after muscle injury [58]. This suggests that fibroblast factors have an important role in muscle regeneration. Therefore, investigating the changes in the skeletal muscle of TCF4 knockout mice with aging might help to partially elucidate the relationship between fibroblasts and muscle aging. Further analysis of the relationship between decreased collagen production and fibroblast dysfunction in aging muscle is needed.

## 4. Materials and Methods

### 4.1. Type of Study

This was an animal experimental study conducted using mice. This study was approved by the Committee of Animal Care and Use of Hokuriku University (approval number, 23-14; approval date, 10 April 2023), and all experimental procedures were conducted in accordance with the institutional guidelines for the use of experimental animals.

### 4.2. Animals

Three-, 10-, 24-, and 28-month-old C57BL/6J male mice (*n* = 24) were obtained from the Jackson Laboratory (Kanagawa, Japan). After purchase, the mice were kept for at least one week before being used as experimental animals. All the animals were housed in individual cages and allowed free access to food and water. The environmental conditions were maintained at 23 ± 2 °C under a 12:12 h light/dark cycle. 

### 4.3. Groups

Mice were divided into four groups according to age (*n* = 6 per group): 3-, 10-, 24-, and 28-month-old mice were assigned to the 3 M, 10 M, 24 M, and 28 M groups, respectively. The number of animals was determined based on previous studies [30,38,59].

### 4.4. Sampling

The mice were weighed and euthanized with cervical dislocation, and the gastrocnemius muscles and soleus muscles were removed and weighed. The right and left muscles were used for molecular and morphological analyses, respectively. Part of the right gastrocnemius muscle and all of the right soleus muscle were excised and preserved in RNAlater (Thermo Fisher Scientific, Waltham, MA, USA), and other parts of the muscles were frozen immediately in isopentane, cooled in dry ice, and stored at −80 °C for further analyses.

### 4.5. qPCR 

Total RNA was extracted from the lateral head of the gastrocnemius and soleus muscles using TRIzol™ reagent (Thermo Fisher Scientific, Waltham, MA, USA). After quality confirmation, the total RNA concentration was normalized to 1 μg, and the RNA was reverse-transcribed to generate first-strand cDNA using random primers and ReverTra Ace (Toyobo, Osaka, Japan). qPCR was performed on a CFX96 Touch Real-Time PCR Detection System (Bio-Rad Laboratories, Hercules, CA, USA) using TB Green Premix Ex Taq II (Takara Bio, Shiga, Japan). The reaction procedure was as follows: one cycle at 95 °C for 30 s, followed by 40 cycles at 95 °C for 5 s and 60 °C for 30 s. A calibration curve was created using the template, and the expression level of each target gene was normalized to that of the housekeeping gene glyceraldehyde-3-phosphate dehydrogenase (*Gapdh*). The target gene expression (upregulation or downregulation) was compared to that of the 3 M group. The primers used for the qPCR were as follows:

*Col1a1*, 5′-GTACTCCTGGTGCTGATG-3′ (Forward) and 5′-GAAGCCTCTTTCTCCTCTCTGA-3′ (Reverse);

*Col3a1*, 5′-CAGGTCCTAGAGGAAACAGA-3′ (Forward) and 5′-TCACCTCCAACTCCAACAATG-3′ (Reverse);

*Col6a1*, 5′-AGGACATCCAGGGCTCCAA-3′ (Forward) and 5′-AGCCTCAAGGCCACACTCTC-3′ (Reverse);

*Col4a1*, 5′-ATGCCAGGAAGAGCAGGAAC-3′ (Forward) and 5′-CGACTACCAGGAAAGCCAACTC-3′ (Reverse);

*Hsp47*, 5′-GCTTGTGAACGCCATGTTCT-3′ (Forward) and 5′-AGGGGCATCTCCACCATCT-3′ (Reverse);

*Mmp13*, 5′-GTGTGGAGTTATGATGATGT-3′ (Forward) and 5′-TGCGATTACTCCAGATACTG-3′ (Reverse);

*Mmp2*, 5′-AAGAAAATGGACCCCGGTTT-3′ (Forward) and 5′-CAACTTCAGGTAATAAGCACCCTTG-3′ (Reverse);

*Mmp9*, 5′-CAGCCAACTATGACCAGGAT-3′ (Forward) and 5′-CTGCCACCAGGAACAGG-3′ (Reverse);

*Gapdh*, 5′-AGGTCGGTGTGAACGGATTTG-3′ (Forward) and 5′-TGTAGACCATGTAGTTGAGGTCA-3′ (Reverse).

### 4.6. Immunohistochemical Analysis

Transverse tissue sections (10-µm thickness) were cut from the middle part of the lateral head of the gastrocnemius and soleus muscles using a cryostat (CM1950; Leica, Wetzlar, Germany) at −25 °C and were mounted on amino silane-coated glass slides. Subsequently, the transverse sections (10-µm thickness) were fixed in 4% paraformaldehyde and rinsed with phosphate-buffered saline (PBS; pH, 7.4). After that, the sections were bleached with 3% H_2_O_2_, rinsed with PBS, and incubated with PBS containing 1% normal goat serum and 0.3% Triton X-100 at 4 °C for 1 h. The sections were then incubated with rabbit polyclonal anti-collagen I, III, IV, or VI antibodies (ab21286, ab7778, ab6586, ab6588; Abcam, Cambridge, MA, USA) diluted to 1:500 in PBS containing 0.3% Triton X-100 at 4 °C for 24 h. Subsequently, the sections were incubated with biotinylated anti-rabbit immunoglobulin G (Vectastain ABC kit; Vector Laboratories, Burlingame, CA, USA) diluted to 1:1000 in PBS for 1 h at 25 °C, followed by incubation with avidin–biotin complex (Vectastain ABC kit) for 1 h at 4 °C. After rinsing with PBS, the sections were washed with Tris-HCl buffer (pH, 7.4) and incubated with diaminobenzidine (0.035%) in Tris-HCl buffer (0.001% H_2_O_2_) for 15 min at 25 °C. After the diaminobenzidine reaction, the sections were stained with hematoxylin, dehydrated using a graded series of ethanol rinses, immersed in xylene, and embedded in Permount™ Mounting Medium (Falma Inc., Tokyo, Japan).

### 4.7. Morphological Analysis

Immunohistochemical staining with an anti-collagen IV antibody was performed to determine the FCSA. The area per image was 393,880 μm^2^; three images were captured randomly (BZ-X800; Keyence, Osaka, Japan), and >150 muscle fibers were analyzed per mouse. Semi-quantitative analysis was performed to detect the intensity of collagen I, III, IV, and VI-IR [60]. The area per image was 393,880 μm^2,^ and three images per gastrocnemius muscle and two images per soleus muscle were analyzed using ImageJ Fiji (Version: 2.14.0/1.54f) [61].

### 4.8. Statistical Analysis

KaleidaGraph statistical software version 4.5.1 (Synergy Software, Reading, PA, USA) was used for statistical analysis. First, all data were expressed as mean ± standard deviation. Next, significant differences between groups were examined using one-way analysis of variance. Means were considered statistically significant at *p* < 0.05. Finally, if there was a significant difference between groups in the one-way analysis of variance, Tukey’s honest post hoc test was used to compare the groups.

## 5. Conclusions

In this study, we examined the effect of aging on collagen gene expression and IR in the skeletal muscle of mice. Aging increased collagen I, III, and VI gene levels and IR in both slow- and fast-twitch muscles and increased collagen IV gene levels and IR in slow-twitch muscle. However, aging decreased the expression of the collagen synthesis marker *Hsp47* in both slow- and fast-twitch muscles but increased the expression of collagen degradation markers. These results suggest that collagen gene expression and accumulation are influenced by muscle fiber and collagen type and that the balance of collagen synthesis and degradation tilts toward degradation with aging. However, the mechanisms by which aging affects collagen synthesis and degradation were not examined in this study, indicating the need for further studies.

## Data Availability

All data are included in the manuscript.
